# Dysregulation of myddosome complex genes its related to alendronate treatment failure in osteoporosis postmenopausal patients

**DOI:** 10.1590/1678-4685-GMB-2025-0070

**Published:** 2026-04-17

**Authors:** Bianca Maria Ribeiro de Oliveira, Maria Julia Alves de Melo, Werbson Lima Guaraná, Camilla Albertina Dantas de Lima, Alexandre Domingues Barbosa, Paula Sandrin-Garcia

**Affiliations:** 1 Universidade Federal de Pernambuco, Programa de Pós-Graduação em Genética e Biologia Molecular, Campus Recife, Recife, PE, Brazil.; 2 Universidade Federal de Pernambuco, Instituto Keizo Asami, Centro de biociências, Campus do Recife, Recife, PE, Brazil.; 3 Universidade Federal de Pernambuco, Departamento de Oceanografia, Centro de Tecnologia e Geociências, Campus Recife, Recife, PE, Brazil.; 4 Universidade Federal de Pernambuco, Hospital das Clínicas, Divisão de Reumatologia, Campus Recife, Recife, PE, Brazil.; 5 Universidade Federal de Pernambuco, Departamento de Genética, Centro de biociências, Campus do Recife, Recife, PE, Brazil.

**Keywords:** Gene expression, bone mineral density, personalized antiresorptive therapy, bisphosphonates

## Abstract

Some patients with osteoporosis (OP) do not respond to treatment with bisphosphonates; pathways that stimulate osteoclatogenesis may be involved in this failure, such as the myddosome pathway. A total of 40 OP patients and 20 controls were included in the group study. Patients treated with sodium alendronate (SA) for two years were classified according to bone mineral density (BMD) variations of the lumbar spine, femoral neck, and total hip, measured by the method of dual-energy x-ray absorptiometry (DXA) as responsive patients (OP-R) (*n* = 20) and non-responders (OP-NR) (*n* = 20), to evaluate the impact of the myddosome pathway gene expression profile in postmenopausal women with OP. The gene expressions were measured through real-time relative quantitative PCR with Taqman^®^ probes; relative quantification was normalized to *GAPDH* and *RPLP0* reference genes. Non-responders showed increased expression levels of *MYD88* and *IRAK3* compared to responders Fold change (FC) = 2.86±1.54, p=0.0002 e FC= 3.62±0.46, p<0.0001 respectively. Our results demonstrate the influence of the myddosome on OP maintenance and response to sodium alendronate (SA) treatment, highlighting the importance of this pathway as a potential target for new therapeutic approaches in postmenopausal OP.

Osteoporosis (OP) is a metabolic and silent disease that affects bone mineral density (BMD), characterized by an increase in the proliferation, differentiation and survival of osteoclasts (OC). These cells are responsible for the resorption of bone tissue and their activity can be modulated by different stimuli such as chemokines, hormones and growth factors. Thus, the resorption of bone tissue can exceed the physiological threshold, generating an imbalance between synthesis and resorption, making the bone trabecula more fragile  (Bhatnagar and Kekatpure 2022; Maeda et al., 2022) .

Bisphosphonates are the most frequently prescribed class of drugs due to their low cost and efficiency in reducing bone fragility. An example of this class of drugs is sodium alendronate (SA). The drug accumulates in resorptive cells and binds to the enzyme farnesyl pyrophosphate synthase, inhibiting the mevalonate pathway and leading to OC apoptosis  (Tonk et al., 2022) .

However, a significant portion of patients treated with SA do not respond or even have an inadequate response to this treatment  (Kim et al., 2023; Yamamoto et al., 2024) . Patients who use the drugs for two years and present a decline in BMD or recurrence of fractures in the same period fall into this case. Osteoporotic patients are already more susceptible to falls because they have lower muscle tone and postural instability. When we observe patients who are not responsive to treatment, there is also a greater propensity to fractures due to continuous bone wear during the period necessary to define the lack of response in these patients. These fractures have a direct impact on an individual’s mobility and are also responsible for high costs to the public health system in terms of hospitalization, medication, surgical costs, and other resources (Copês et al., 2021).

Treatment failure can be attributed to several factors, such as duration and adherence to treatment, causes secondary to osteoporosis, and genetic and immune factors contributing to the maturation and proliferation of OC, such as an increase in inflammatory cytokines (Vega et al., 2023).

In this context, the myeloid differentiation primary response protein pathway (MYD88) may interfere with this treatment, since the onset of its signaling is given by toll-like receptor 4 (TLR4) and interleukin receptor 1 (IL-1R), both of which have a function related to osteoclastogenesis and increased survival of OC (Duan et al., 2022; Jalal Alshaweesh et al., 2024). The canonical pathway of MYD88 (Figure 1) makes use of the TNF-associated receptor associated with factor 6 (TRAF6) as an adaptor protein, culminating in the release of the effector part of nuclear factor kappa beta (NF-kB1) to the nucleus, where it activates inflammation-related transcription factors, such as interleukin 1β (IL-1β) and interleukin 6, in addition to promoting the inhibition of osteoblastogenic factors, such as the transcription factor of the RUNX2 family (RUNX2) (Rojasawasthien et al., 2022). On the other hand, Interleukin 3 receptor associated kinase (IRAK3) acts as a pathway regulator by promoting dissociation between TRAF6 and the other components of the signaling cascade, thereby preventing its activation (Gomes da Silva et al., 2021). In bone metabolism, hyperactivation of the MYD88 pathway promotes osteoclastogenesis and inflammatory bone loss, whereas loss or inhibition signaling impairs osteoblast differentiation and alters bone formation and remodeling (Jalal Alshaweesh *et al.,* 2024; Lainšček et al., 2024).

**Figure 1-:**
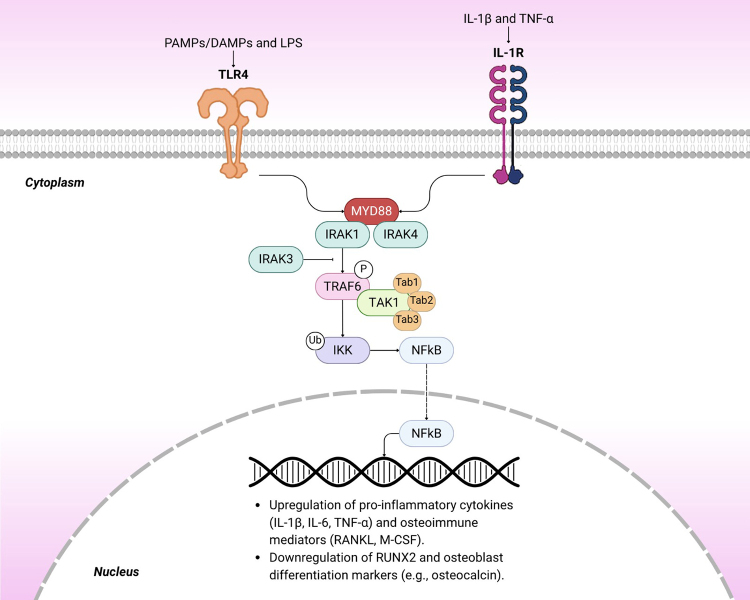
Schematic representation of the MyD88-dependent NF-κB signaling cascade activated through TLR4 and IL-1R. Extracellular inflammatory stimuli such as PAMPs/DAMPs, LPS, IL-1β, and TNF-α induce receptor engagement and MyD88 recruitment, promoting myddosome assembly and downstream activation of TRAF6 and the IKK complex. Phosphorylation and degradation of IκB release NF-κB, which translocates to the nucleus and promotes the transcription of pro-inflammatory cytokines (IL-1β, IL-6, TNF-α) and osteoimmune mediators (RANKL, M-CSF), while repressing RUNX2 and osteoblast differentiation markers (e.g., osteocalcin). These events contribute to a pro-inflammatory and catabolic bone microenvironment.

Therefore, the present study evaluated the influence of the MYD88 canonical pathway on OP and its role in patients’ non-responsiveness to bisphosphonate treatment in a population of postmenopausal women.

For the study, patients with postmenopausal OP were selected at the clinical outpatient clinic of Rheumatology of the Hospital of the Federal University of Pernambuco (HC-UFPE). The diagnosis of postmenopausal OP was based on the criteria of the World Health Organization (WHO). Considering the T-Score of the bone densitometry test (g/cm^2^). The inclusion criteria were having at least two years of treatment with SA, with oral doses of 70 mg/week, and residing in the state of Pernambuco. Women with osteopenia and women affected by other rheumatic and/or inflammatory diseases, in addition to those who used other medications for bone diseases, were excluded from the study.

The group of patients included (N = 40) was divided according to the response to treatment, being classified as non-responsive OP (OP-NR) (N = 20) based on bone densitometry tests, where they presented, for at least 2 years a decline in BMD when compared to previous tests. The group of OP-responsive patients (OP-R) (N = 20) was composed of patients who presented BMD stabilization or increase after two years of treatment (Gani et al., 2023). The evaluation was performed using BMD parameters in the lumbar spine region (LS) (L1-L4) and total hip (TH), with the least significant change (LS = 0.051 g/cm^2^; TH = 0.033 g/cm^2^), thus values below those described were classified as non-response to therapy (Guaraná et al., 2024).

The control group was made up of postmenopausal women (N = 20, 60.6 ± 5.51 years) who did not have a diagnosis of OP or osteopenia, according to dual-energy x-ray absorptiometry (DXA), in addition to a negative history of fractures. The study excluded patients with a history of cancer, diabetes and other rheumatological diseases. 

Clinical data and blood (10 ml) were collected for both the control group and the patients at consultation. The following were evaluated: age at menarche (years), age at menopause (years), time at menopause (years) 25-hydroxyvitamin D (ng/mL), calcium (mg/dL), alkaline phosphatase (ALP; U/L), phosphorus (mg/dL), and parathyroid hormone (PTH; pg/mL).

The Research Ethics Committee of the Health Sciences Center of the Federal University of Pernambuco (CEP/CCS/UFPE, protocol number 513/11) approved the entire study. All participants provided written consent to participate in the study and to provide their medical records.

RNA was extracted from the peripheral blood of patients and healthy controls using TRIzol^®^ (Invitrogen, Carlsbad, CA, USA). The cDNA was performed by the GoScript™ kit (Promega, Madison, WI, USA) was used, following the manufacturer’s instructions.

Quantitative real-time PCR (qRT-PCR) was performed in technical triplicate using Taqman^®^ expression probes for all specific target genes *MYD88, IRAK3, TRAF6*, and *NF-kB1* (Thermo Fisher Scientific, California, USA). The reactions were performed in the ABI 7500 Real-Time PCR detection system (Thermo Fisher Scientific, California, USA). For the normalization of the relative quantity (RQ), the *GAPDH* and *RPLP0* genes (TaqMan^®^ probes) were used as endogenous controls (Thermo Fisher Scientific, California, USA) (Adeola, 2018). RQ of mRNA of the genes was measured from the quantification cycle (Cq) of the target and reference gene. The relative expression values were calculated according to the MIQE guidelines (Bustin et al., 2025). This method involves calculating the geometric mean of the RQ values of the reference genes to obtain the normalization factor (NF). Next, the Relative Quantity Normalized (RQN) was obtained by dividing the RQ of the target gene by the NF. Fold Change (FC) values were obtained by dividing the RQ of a sample by the mean RQN of the control samples.

The data obtained were submitted to the Kolmogorov-Smirnov test to assess the normality of the data. The difference between the groups was analyzed using the Student’s t-test or the Mann-Whitney test for parametric and non-parametric data, respectively. All analyses were performed using the GraphPadPrism^®^ software, version 8.0 (GraphPad Software, San Diego, CA, USA), considering p < 0.05 significantly statistical.

Clinical and laboratory data of the patients included in the study were compared to the healthy control group. The mean age (OP = 69.49 ± 8.3 years; control = 60.5 ± 5.34 years) and time since menopause (OP = 27.17 ± 10.84; control = 14.06 ± 9.74 years) were higher in the patient than in the control group (p < 0.001). Other clinical data such as menarche, age at menopause, and laboratory data such as calcium, vitamin D, alkaline phosphatase, parathyroid hormone, and phosphorus did not show statistically significant differences (p > 0.05). (Table 1).

Regarding the response to treatment, the patients when comparing OP-R and OP-NR, no statistical difference was observed in any of the data analyzed between the groups, except for the mean age (OP-R= 72.15 ± 7.49; OP-NR: 66.66 ± 7.58), which was higher in responsive patients (p = 0.003) (Table 1).

**Table 1-:** Clinical and laboratory analysis of the group of patients and healthy controls.

Variables	CT (N=20)	OP (N=40)	*P value*	OP-R (N=20)	OP-NR (N=20)	*P value*
Age (years)	60.5 ± 5.34	69.49 ± 8.3	0.0001*	72.15 ± 7.49	66.66 ± 7.58	0.003*
Mean of age at menarche (years)	13.56 ± 2.02	14.27 ± 1.95	0.245	14.38 ± 2.3	14.13 ± 1.5	0.94
Mean of age at menopause (years)	45.4 ± 8.81	44.15 ± 7.62	0.61	42.76 ± 7.3	45.62 ± 6.60	0.33
Mean of menopause duration (years)	14.06 ± 9.74	27.17 ± 10.87	0.0001*	29.64 ± 9.6	24.7 ± 11.74	0.18
Lumbar Spine (L1-L4) (g/cm2)				0.74 ± 0.14	0.68 ± 0.09	0.06
Calcium (mg/dL)	9.2 ± 0.49	9.46 ± 0.46	0.169	9.57 ± 0.15	9.54 ± 0.55	0.85
25- hydroxyvitamin D (ng/mL)	28.8 ± 6.88	28.5 ± 7.58	0.935	25.43 ± 6.75	31.95 ± 6.18	0.56
PTH (pg/mL)	54.51 ± 31.33	60.11 ± 27.79	0.62	61 ± 27.36	74.71 ± 54.04	0.52
Phosphorus (mg/dL)	3.5 ± 0.50	3.41 ± 0.6	0.69	3.32 ± 0.63	3.71 ± 0.41	0.19
ALP (U/L)	89.07 ± 42.24	72.4 ± 22.1	0.39	92 ± 73.48	76.8 ± 35.23	0.60

Descriptive analyses were expressed as mean and standard deviation. * Indicate values with statistical significance (*p* < 0.05) by Mann-Whitney U-test. OP: osteoporotic patients; CT: healthy controls; PTH: parathyroid hormon; ALP: alkaline phosphatases; OP-R: responsive patients; OP-NR: Non-responder.

In the evaluation of myddosome gene expression, it is possible to observe that *MYD88* and *IRAK3* showed increased expression in the group of patients, although the data did not present statistical significance (FC = 1.42 p = 0.345) and (FC = 1.85 p = 0.33) respectively (Figure 2 A-B). In addition, the OP group showed a significant increase in *TRAF6* expression (FC= 6.47 p < 0.0001) (Figure 2 C), while *NF-Kb1* was less expressed in the same group (FC= 0.5 p < 0.0001) (Figure 2 D) when compared to the control group.

The differential expression of myddosome genes was also evaluated according to the response to treatment: OP-R and OP-NR patients.

The *MYD88* and *IRAK3* genes showed a significant increase in the OP-NR group (FC = 2.86, p = 0.002) (Figure 2E), and (FC= 3.62, p < 0.0001), (Figure 2 F), respectively. The *TRAF6* and *NF-kB1* genes were not statistically significant in the analysis (FC= 0.82, p= 0.346) and (FC= 1.15, p= 0.297) (Figure 2 G-H).

**Figure 2-:**
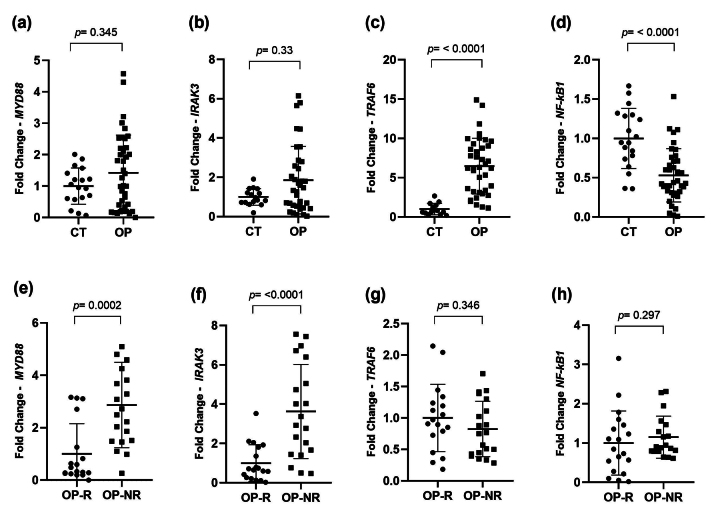
Levels of expression of myddosome genes in the osteoporosis (OP), healthy control (CT), responsive patients (OP-R) and non-responder (OP-NR) patient groups. In the analysis of relative expression between the OP and CT groups, patients exhibited higher expression of the *TRAF6* gene (fold change (FC) = 6.47, p < 0.0001) (c), and lower expression of *NF-κB1* (FC = 0.5, p < 0.0001) (d). The comparative analysis of *MYD88* (a) and *IRAK3* (b) between the OP and CT groups was not statistically significant (p > 0.05). When the OP-R and OP-NR groups were compared, the OP-NR group showed significantly higher expression of the *MYD88* (e) and *IRAK3* (f) genes (FC = 2.86, p = 0.0002 and FC = 3.62, p < 0.0001, respectively). Meanwhile, the *TRAF6* (g) and *NF-κB1* (h) genes were not statistically significant. All analyses were performed using the Mann-Whitney test.

The present study evaluated the expression of myddosome complex genes concerning the development of OP and their influence on the response to treatment with SA. This study examined how the expression of myddosome complex genes influences the development of osteoporosis (OP) and the response to treatment with SA. A significant difference in age was observed between the groups in the clinical analysis. While the OP group was significantly older than the CT group (p < 0.001), this reflects the natural epidemiology of postmenopausal osteoporosis rather than being a confounding factor. While chronological ageing is associated with gradual reductions in bone turnover, OP results from oestrogen deficiency-driven inflammatory activation of osteoclastogenic pathways, including MyD88/NF-κB and RANKL signalling (Bhatnagar and Kekatpure, 2022).

The gene expression analysis performed comparing the control group and patients with OP showed a considerable increase in *TRAF6* expression (FC= 6.47 p < 0.0001) and a slight decrease in *NF-kB1* expression levels (FC= 0.5 p < 0.0001) in the patient group. The relationship between *TRAF6* and osteoporosis may be due mainly to the activation of the RANK/RANKL pathway, the released ligand binds to its receptors on the resorptive cells, recruiting TRAF6 as an adaptor protein stimulating OC differentiation through the expression of genes such as nuclear factor of activated T cells 1 (NFATc1), which functions as a co-stimulator in OC differentiation (Li et al., 2024).

However, the results observed in the expression of these two genes may suggest the activation of a pathway mediated by *TRAF6* without the action of *NF-kB1*, such as the Janus kinase (JNK) pathway, extracellular signal-regulated kinase (ERK) and protein kinase B (AKT), which can stimulate the formation of mature OC (Zhi et al., 2020). Furthermore, all the patients included had undergone at least 2 years of treatment with SA, which may also have interfered with *NF-kB1* levels in this group. A study by Feng Xin et al. (2020) observed similar data in mice with osteoarthritis treated with platelet-rich plasma and SA, obtaining a reduction in the protein expression of p-p65/p65 and IκBα (FC = 2 p< 0.01). This result was even more significant than when platelet-rich plasma treatment alone was used, demonstrating the potentiating effect of SA on this pathway (Xin *et al.,* 2020). 

Zhang et al. (2025) found the same response profile when treating mice with periodontitis with nanoparticles containing SA, which resulted in reduced bone resorption and increased bone volume. RNA-seq analyses revealed significant variations in the NF-κB pathway, and in vitro assays confirmed reduced NF-κB gene and protein expression in the treated group compared to the control group. Additionally, the treated group exhibited reduced p65 phosphorylation, a critical step in NF-κB activation. Therefore, the decrease in p-p65 phosphorylation corresponds to *NF-κB* inhibition and the inability of *NF-κB* to translocate to the nucleus. (Zhang *et al.,* 2025).

When the gene expression profile of the patients was evaluated in relation to their response to treatment, a significant increase in the expression of *MYD88* (FC = 2.86 ± 1.54 p = 0.002) and *IRAK3* (FC = 3.62 ± 0.46 p < 0.0001) was observed in the group of patients who did not respond to treatment, suggesting an involvement of this pathway in the failure of treatment with SA. The influence of OP and SA on the myddosome pathway has already been demonstrated for Vijayan et al. (2013) in mice with induced menopause, submitted to methionine treatment to inhibit bone loss. This effect has been demonstrated *in vitro* by culturing OC, where treatment with methionine resulted in a protein decrease in TLR4, MyD88, and NF-κB. This decrease was even more pronounced with the addition of SA to the methionine treatment, all the myddosome pathway expression data under the effect of methionine and SA obtained p < 0.05. Thus, positive modulation of the pathway directly affects bone wear, while an effective treatment for reducing bone fragility involves its inhibition (Vijayan *et al.,* 2013). In addition, the silencing of *MYD88* by siRNA has already been described as responsible for inhibiting chemotactic molecules and reducing osteoclastogenesis *in vitro*, demonstrating its fundamental role in the differentiation of OC (Vijayan *et al.,* 2013). Thus, the increase in the expression of this gene is related to an increase in the quantity of OC, causing continuous resorption and possible refractoriness to treatment.

In contrast, the increase in *MYD88* expression could suggest a decrease in *IRAK3* mRNA levels as it is a regulatory molecule since non-responsiveness to treatment would be explained by *MYD88* overstimulation of the pathway and lack of inhibition due to the absence of *IRAK3*, which would cause IκBα degradation and consequently the transformation of NF-kB1 into active dimers, increasing its activity. This relationship is also seen in other inflammatory diseases, such as rheumatoid arthritis, where the expression of *IRAK3* is inversely proportional to that of *MYD88*, so an increase in *IRAK3* corresponds to a decrease in NF-κB1 signaling (Gomes da Silva et al., 2021). However, in our results, we observed an increase in *IRAK3* in OP-NR patients, which could be explained by a rebound production to the overstimulation of *MYD88*. In a study carried out by Norton et al. (2011), IRAK3 showed the same protein expression profile in PBMC cell cultures from non-responsive patients treated with zoledronic acid, another nitrogen bisphosphonate, with its expression levels remaining high in cells from non-responsive patients when compared to cell cultures from responsive patients. In the same study, macrophages derived from mice and submitted to the same treatment were also evaluated, and a decrease in IRAK3 protein levels was observed, with no change in the level of mRNA, pointing to the involvement of post-transcriptional mechanisms; however, these data were not statistically significant (Norton *et al.,* 2011). However, the increased expression of *IRAK3* in the non-responder patients in our study may be correlated with post-transcriptional alterations, where the increased expression of *IRAK3* may not culminate in its translation so the pathway is not regulated and generates a high rate of OC proliferation and cytokine release (Tao et al., 2021). 

We have considered the points mentioned above, as well as the importance of our findings on the deregulation of the myddosome pathway through *IRAK3*. Despite high levels of expression, the pathway is not suppressed. For this reason, further research is required to investigate the mechanisms of IRAK3 action. Some miRNAs, such as miR-33b-3p and MiR-539-5p, are reported to target IRAK3, with an inverse correlation being reported in the expression levels of *IRAK3* and miRNAs (Hu and Miao, 2021; Tao et al., 2021). However, none of these studies have investigated the interaction between miRNAs and IRAK3 in relation to OP. 

According to the gene expression profile of non-responders, the lack of response to AS treatment is due to an exacerbation of the pathway and failure of the regulatory component. Therefore, these patients would have hyperactive osteoclastogenic pathways and an increased number of resorptive cells, even when AS is used. This is why there is no recovery of BMD even after two years of treatment.

In conclusion, our findings suggest the influence of differential expression of the myddosome pathway on the maintenance of OP and that exacerbation of its activity may be a contributing factor to treatment failure with SA. However, the study has some limitations, such as the limited number of patients due to the difficulty in obtaining patient follow-up data for at least 2 years. Finally, future analyses should include a larger number of patients and additional analyses to make the results more robust.

## Data Availability

The data that support the findings of this study are available from the corresponding author upon reasonable request.
